# Avoidance of different durations, colours and intensities of artificial light by adult seabirds

**DOI:** 10.1038/s41598-021-97986-x

**Published:** 2021-09-23

**Authors:** Martyna Syposz, Oliver Padget, Jay Willis, Benjamin M. Van Doren, Natasha Gillies, Annette L. Fayet, Matt J. Wood, Aarón Alejo, Tim Guilford

**Affiliations:** 1 Department of Zoology, University of Oxford, Mansfield Road, Oxford, OX1 3SZ UK; 2 Cornell Lab of Ornithology, Cornell University, Ithaca, NY 14850 USA; 3 School of Natural & Social Sciences, University of Gloucestershire, Francis Close Hall, Cheltenham, GL50 4AZ UK; 4 Department of Physics, University of Oxford, South Parks Road, Oxford, OX1 3PS UK

**Keywords:** Animal behaviour, Conservation biology

## Abstract

There is increasing evidence for impacts of light pollution on the physiology and behaviour of wild animals. Nocturnally active Procellariiform seabirds are often found grounded in areas polluted by light and struggle to take to the air again without human intervention. Hence, understanding their responses to different wavelengths and intensities of light is urgently needed to inform mitigation measures. Here, we demonstrate how different light characteristics can affect the nocturnal flight of Manx shearwaters *Puffinus puffinus* by experimentally introducing lights at a colony subject to low levels of light pollution due to passing ships and coastal developments. The density of birds in flight above the colony was measured using a thermal imaging camera. We compared number of flying shearwaters under dark conditions and in response to an artificially introduced light, and observed fewer birds in flight during ‘light-on’ periods, suggesting that adult shearwaters were repelled by the light. This effect was stronger with higher light intensity, increasing duration of ‘light-on’ periods and with green and blue compared to red light. Thus, we recommend lower light intensity, red colour, and shorter duration of ‘light-on’ periods as mitigation measures to reduce the effects of light at breeding colonies and in their vicinity.

## Introduction

Ecological light pollution has been defined as “artificial light that alters the natural patterns of light and dark in ecosystems”^1^. Artificial light has changed the natural day-night regime across almost a quarter of the globe and is increasing by 2% every year^2, 3^. Over the last decade, there has been a rapid increase in empirical studies on the effects of light pollution on animals, encompassing organismal physiology, phenology, onset of activities, life history traits such as size, cognition or predation risk, and abundance and diversity of populations^4^. While there are some examples of positive influences of artificial light on individual fitness, such as bats depredating easier-to-detect insects^5^, there is mounting evidence of detrimental effects of artificial light on animals^4^.

Interestingly, and of relevance to potential mitigation measures for the negative effects of artificial light, the extent to which animals respond to artificial light has been found to vary with the duration, intensity and spectrum of light^6–10^. Lowering light intensity and using intermittent lights results in fewer affected animals across different study sites^6, 7, 10–14^. For example, Mrosovsky^15^ found that longer ‘light-off’ periods can decrease the preference of sea turtles for lit areas. Furthermore, the usage of different wavelengths also reduces the negative effect of light pollution on animals. These findings, however, might depend on species’ visual systems, as studies investigating birds’ attraction variously recommend either broadband white light^8^, short (green^16^ but see^17^), or long wavelengths (red and yellow^9, 10, 18^) for reducing numbers of stranded birds. So far, however, longer wavelengths (red and yellow), seem to attract fewer insects^19^ and sea turtles^20^, and cause less disturbance to the movements of slow-flying bats, such as *Plecotus* and *Myotis*^5^. Slow-flying bats are negatively phototactic as a way of reducing their predation risk^21^, thus artificial light created by streetlamps and other sources changes and restricts their movement, contributing to population declines^22^. Positive phototaxis, unlike negative phototaxis, is easier to detect since it results in congregations of animals around anthropogenic light sources. It directly impacts animal populations, such as sea turtles, some groups of insects, and birds, by causing fatal collisions with light-emitting objects (e.g. streetlamps, buildings, radio towers) or resulting in animals not being able to leave artificially lit areas, either due to misleading navigational cues and/or obstructions^23–27^. Such areas can be poor in food resources, or have a higher exposure to predators, thus aggravating the negative effects of artificial light^23, 25, 28^.

Attraction to light is especially common in birds, causing millions of casualties annually due to collisions with illuminated structures, including lighthouses^29^, broadcast towers^30^, wind turbines^31^ and buildings in towns^25^. Although many birds are affected by light pollution, burrow-nesting seabirds from the Procellariiformes order are commonly found to land in lit areas—a phenomenon known as “fallout” or “grounding”^32^. These birds cannot easily become airborne from built-up areas due to their forward-heavy anatomy, which is adapted to life at sea^33^. They require a slope, a gust of wind or a long runway to take off, but cars and buildings form a barrier, and so groundings can often be fatal^14^. Research evaluating the factors affecting the severity of fallout reports that the full moon can create enough ambient background light to lessen the negative effect of artificial light on seabirds and decrease the number of grounded birds^34–36^. A study investigating the influence of wavelength composition on seabird fallout found that filtering out short wavelengths up to 470 nm (leaving the spectral range of colours between green, yellow, and red) did not result in a decrease in the number of grounded Newell’s shearwaters (*Puffinus newellii*)^37^. A report on tropical shearwaters (*Puffinus bailloni*) revealed, however, greater attraction in this species to blue and green colours than red and yellow^38^. Furthermore, Rodríguez et al.^39^ found that grounding of short-tailed shearwaters (*Ardenna tenuirostris*) was reduced with high-pressure sodium streetlamps, which contain less blue light than metal halide and light emitting diode (LED) lamps. These findings are supported by an examination of the retina of wedge-tailed shearwaters (*Puffinus pacificus*), which indicates greater sensitivity to short (blue and green) wavelengths than to longer wavelengths (red)^40^. Most studies, however, have focused on the effects of light pollution in urban areas where birds ground during the fledgling season^25, 37, 39^. Light pollution also occurs at or near the breeding grounds in the form of lit-up buildings, structures or handheld lights used by visitors to observe seabirds or walk around a colony, but how this may affect adult seabirds’ behaviour remains poorly understood.

**Figure 1:**
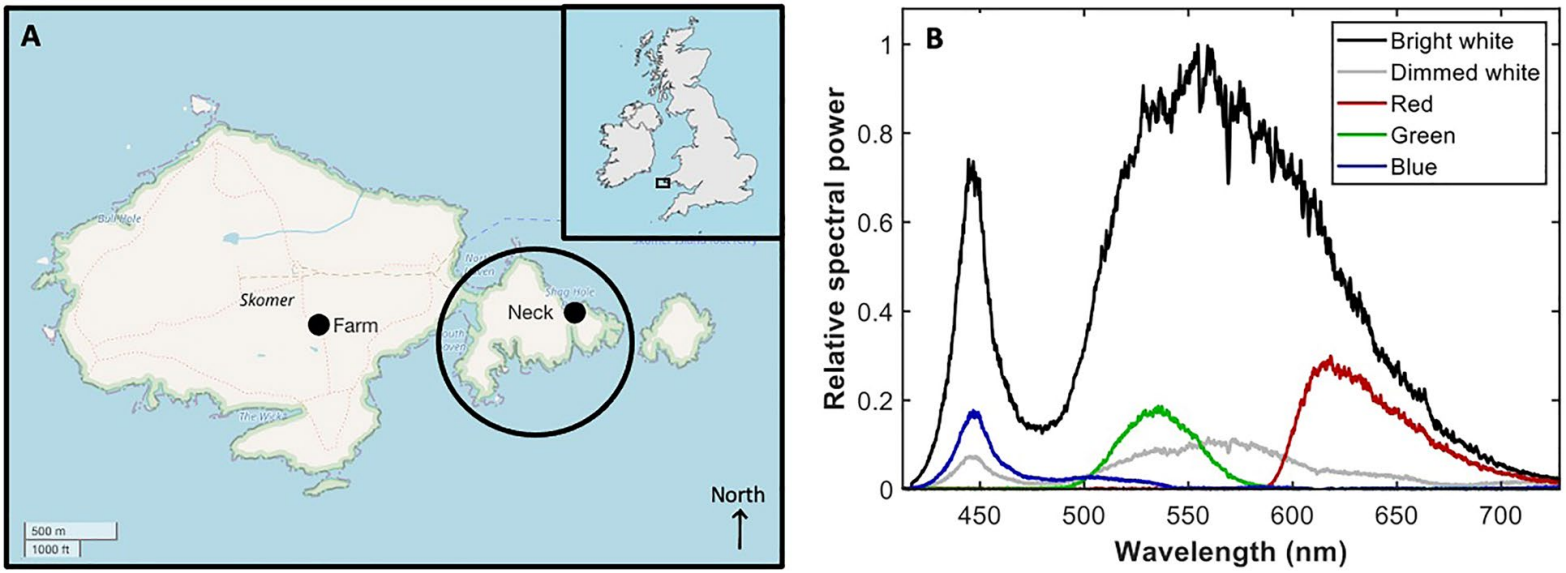
(**A**) Map of Skomer Island indicating the locations of the experiment on the Neck (right) and near to the farmhouse (the Farm, left) using black dots. The area that tourists are not allowed to access is encircled in black (OpenStreetMap, 2020). (**B**) Spectra of the light sources used in the experiment showing wavelength-dependent intensity of the light source employed depending on the filter in place to produce the light sources required; namely red, green, blue, dimmed white, and bright white. The flux for the torch covered with the red (4.3 W), green (2.0 W), and blue (1.4 W) filter as well as for dimmed white (1.4 W) was of the same order of magnitude, with an average value of (2.7 ± 1.3) W. The flux for the torch without a filter was ten times greater (32 W).

Here, we investigate the relative effects of differing durations, colours and intensities of light on the number of Manx shearwaters (*Puffinus puffinus*) observed in flight at their breeding colony. Similar to many other Procellariiformes, Manx shearwaters forage at sea during the day but visit their nests only at night, probably to avoid predation^41–44^. We monitored the nocturnal movements of adult shearwaters in a dense breeding colony, at two separate sites subject to different levels of human disturbance (Fig. 1), and in response to experimentally altered light conditions. We used supervised machine learning software to count the number of shearwaters passing through the field of view of a thermal camera. Our study comprised two experiments, which examined how the number of flying shearwaters changed in response to (1) light intensity and spectral wavelength (spectra experiment) and (2) light intensity and duration of exposure (interval experiment). A recent study by Guilford et al.^45^ found that Manx shearwaters are more likely to collide with an illuminated structure than a dark one in foggy conditions, suggesting shearwaters may be attracted to light (positive phototaxis). However, conflicting evidence suggests that adult Procellariiform seabirds might actually be repelled by light (negative phototaxis). These include observations of a smaller proportion of grounded adult shearwaters compared to juveniles during fallouts^35, 36^, as well as findings that chick provisioning visits by Scopoli’s shearwaters (*Calonectris diomedae*) decreased during a disco event^46^. In the latter experiment, no effect was reported on moonlit nights, highlighting the importance of seabirds’ visual perception when interpreting responses to light pollution. Thus, whilst there is clear evidence that artificial light does affect the behaviour of seabirds such as shearwaters, and that this may depend on the background natural light regime or general visibility, it is less clear whether adult Manx shearwaters would be expected to respond with negative or positive phototaxis to artificial light. We attempted to resolve this issue using our experimental set-up. Firstly, we compared the difference in the number of birds counted during ‘light-on’ versus ‘light-off’ periods. An increase in the number of birds counted in ‘light-on’ compared to ‘light-off’ periods would indicate positive phototaxis, whereas a decrease would indicate negative phototaxis. We next investigated covariates of the strength of this response. Previous research indicates that the responsiveness of birds to light is stronger under increased light intensities and durations^6, 7, 11–14^. Therefore, regardless of the direction of shearwaters’ response to light, we expected that the magnitude of this effect would increase with intensity and duration. Furthermore, we expected that natural background light created by the moon and other celestial objects might reduce the effect of the experimentally-introduced light pollution^34–36^ and thus, result in more (negative phototaxis) or fewer (positive phototaxis) flying birds during a full moon compared to a new moon under the same experimental conditions. Finally, as two closely related shearwater species have been reported to exhibit greater sensitivity to lower wavelengths of light (more blue), we expected to observe weaker responses to light with longer wavelengths (more red)^37–40^. Thus, we hypothesised that we would observe fewer (positive phototaxis) or more (negative phototaxis) flying seabirds under red light compared to green and blue.

## Results

We carried out two experiments to investigate the effect of different light characteristics on the number of flying Manx shearwaters counted passing through the field of view of a thermal camera at night. We recorded a total 47 h 28 min of video footage. The spectra experiment (17 h 30 min of video footage) assessed the effect of different wavelengths and intensities, whereas the interval experiment (29 h 58 min) explored the effect of different intensities and duration of treatments on the number of flying seabirds. The counts of flying birds were performed using a Motion-Based Multiple Object Tracking module in MATLAB (R2017a, MathWorks Inc.), which tracks moving objects in two dimensions. We validated the method by comparing counts of birds returned by the module (supervised machine learning) and those counted manually and found that they were well correlated (Pearson’s Correlation test sample estimates 96.72% ± 0.93%, t_238_ = 58.791, *P* < 0.001, Supplementary Fig. S1). Consequently, we used the counts obtained through machine learning as the experimental measurement throughout all the video sequences (including those sections of video used as test periods for manual selection). This choice produced an objective, reproducible method of measurement without bias through human intervention in selection.

### The spectra experiment

**Figure 2:**
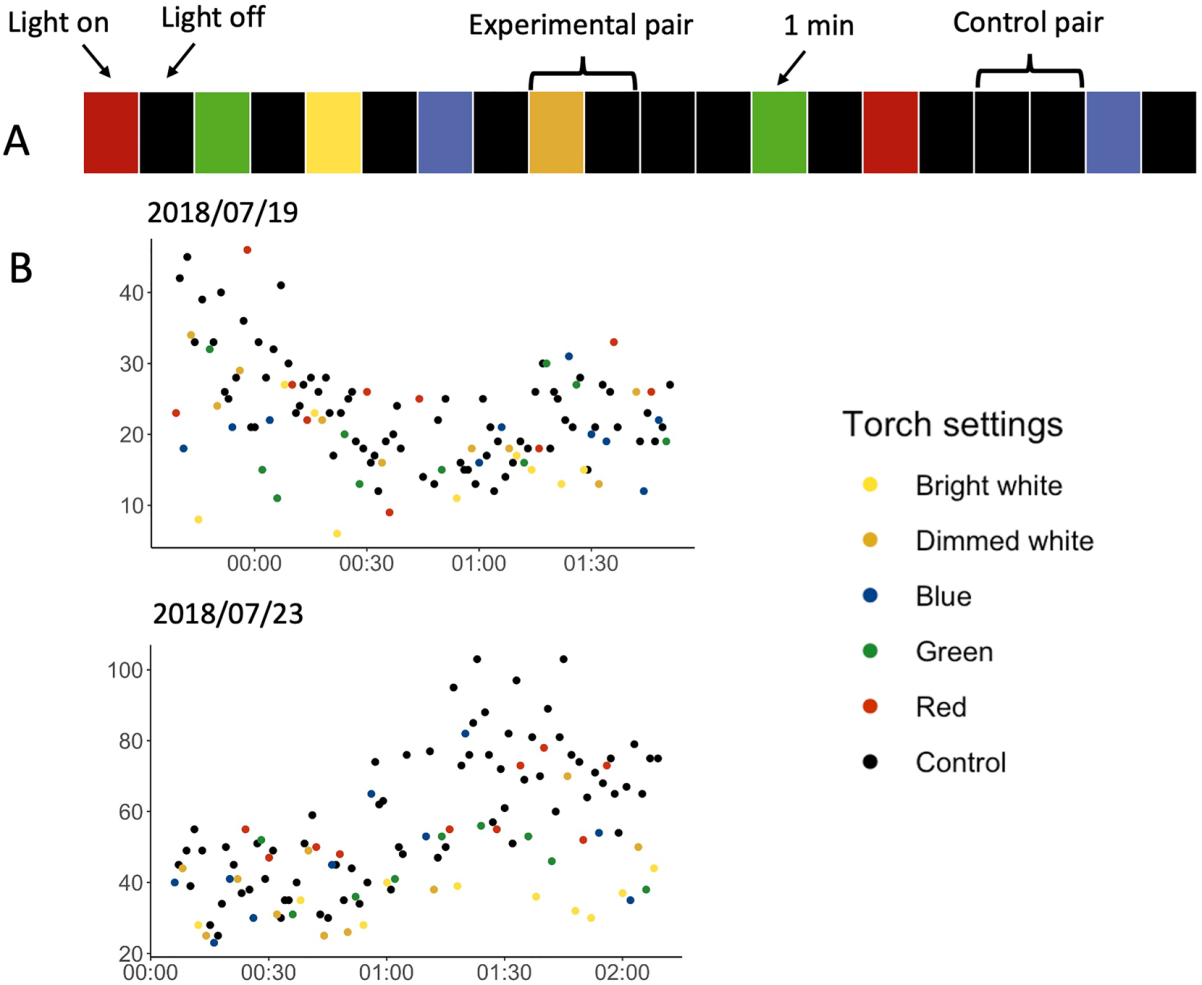
(**A**) Visualisation of an example of treatment series of the spectra experiment, with consecutive 1-min segments (rectangles on the figure) with the torch on and off. Each experimental pair (‘light-on’, ‘light-off’) has a coloured segment corresponding to one of five different settings (‘red’, ‘green’, ‘blue’, ‘dimmed white’ and ‘bright white’) followed by a black segment representing the light off. Two black segments indicate a control pair (‘light-off’, ‘light-off’). (**B**) Two example days of the spectra experiment undertaken at the Farm (19th July 2018 starting at 23:40BST) and on the Neck (23rd July 2018 at 00:07BST). They show the count of birds per minute against time. Different colours of dots represent various settings.

**Figure 3:**
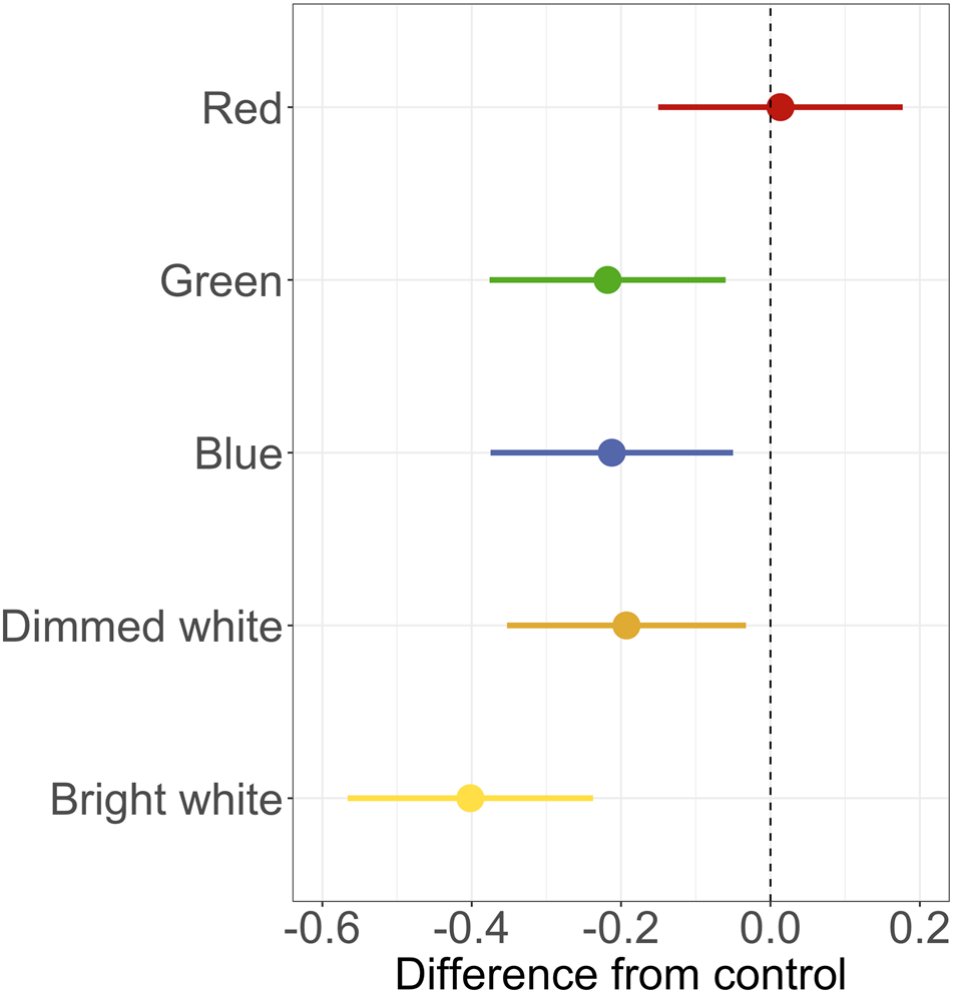
Results of post-hoc tests in the spectra experiment. The graph shows the estimated coefficients and 95% confidence interval of five experimental pairs when comparing the difference in ‘light-on’ vs ‘light-off’ to the control (‘light-off’ vs ‘light-off’, dashed vertical line). All the colours except red significantly reduced bird counts, with lower numbers of birds recorded when the light was on. For detailed statistics, see Table 1.

We conducted this experiment in two-minute trials. Each experimental pair consisted of turning a light on for one minute and subsequently turning it off for one minute. For control pairs, we kept the light off for two consecutive minutes (Fig. 2A). This resulted in 87 bright white, 87 dimmed white, 85 blue, 88 green, and 81 red experimental pairs, and 86 paired control pairs. To investigate how different light colours affected the number of flying shearwaters, we compared the difference in counted birds in the experimental pairs (‘light-on’ vs. ‘light-off’) to the difference in the control pairs (‘light-off’ vs. ‘light-off’) using a post-hoc generalized additive model (GAM). The GAM was well fitted, with 92.9% of deviance explained. Exposure to all colours except red resulted in a significantly lower bird count compared to the control (Table 1, Figs. 2B, 3). Bright white light had the strongest effect, causing a 33% (95% CI [24, 40]) reduction in counted birds compared to the control pair (‘light-off’, ‘light-off’). Dimmed white, blue and green colours were not significantly different from each other, causing a similar effect (decreases of 18% (95% CI [8, 27]), 19.1% [9, 28], and 20% [10, 28], respectively). Exposure to red light had no significant effect on the number of flying birds and this treatment was significantly different from all the others (Table 1).

**Table 1:** Summary of the results of post-hoc pairwise tests of the comparison of differences in bird counts between experimental pairs (‘light-on’, ‘light-off’) and control pairs (‘light-off’, ‘light-off’).

Comparisons	Estimate	Odds Ratio	Std. Error	t ratio	*P*-value
**Control vs Bright white**	**− 0.396**	**0.673**	**0.06**	**− 6.58**	< 0.0001***
**Control vs Dimmed white**	**− 0.198**	**0.82**	**0.059**	**− 3.375**	0.0102**
**Control vs Blue**	**− 0.212**	**0.809**	**0.059**	**− 3.571**	0.0051**
**Control vs Green**	**− 0.218**	**0.804**	**0.058**	**− 3.77**	0.0025**
Control vs Red	0.018	1.018	0.06	0.296	0.9997
**Bright white vs Dimmed white**	**0.198**	**1.219**	**0.06**	**3.295**	0.0132**
**Bright white vs Blue**	**0.183**	**1.201**	**0.061**	**3.01**	0.0324*
**Bright white vs Green**	**0.178**	**1.195**	**0.059**	**2.99**	0.0343*
**Bright white vs Red**	**0.414**	**1.513**	**0.061**	**6.735**	< 0.0001***
Dimmed white vs Blue	**− **0.015	0.985	0.059	**− **0.246	0.9999
Dimmed white vs Green	**− **0.02	0.98	0.058	**− **0.353	0.9993
**Dimmed white vs Red**	**0.216**	**1.241**	**0.06**	**3.598**	0.0047**
Blue vs Green	**− **0.006	0.994	0.059	**− **0.099	1
**Blue vs Red**	**0.23**	**1.259**	**0.061**	**3.788**	0.0023**
**Green vs Red**	**0.236**	**1.266**	**0.059**	**3.986**	0.0011**

Negative estimates indicate that bird numbers decreased in the presence of illumination compared to control periods. Estimates taken from the GAM (spectra experiment) show log-transformed differences in counted birds. For example, bright white caused a (0.67 – 1) * 100% = -33% decrease in counted birds when we turned on the light compared to control pair (‘light-off’, ‘light-off’). Significant results are marked in bold. ***P < 0.001; **P < 0.01; *P < 0.05.

The spectra experiment was undertaken at two locations on Skomer Island, one near to the Farm, where overnight guests and staff reside, and another on the Neck, a minimum access area that is off-limits to tourists (Fig. 1A). Birds reacted similarly to the light treatments at both sites (Supplementary Table S1). For the red light treatment only, we observed an effect of ambient brightness on the numbers of observed shearwaters (Supplementary Table S2): for every one-unit increase (representing one standard deviation) in night darkness, there was a 22.2% (95% CI [6, 35]) increase in the effect of red light on shearwater counts. In other words, we counted fewer shearwaters in red light treatments during dark nights compared to moonlit nights. The smoothed terms of time relative to midnight, as well as the random effects of ‘pair’ (paired on/off lights) and calendar day showed significant effects on the numbers of birds counted, implying that there was variation owing to factors influencing fluctuation in colony attendance during the night, which we accounted for in our model (Supplementary Table S3). Changes in colony attendance are caused by weather conditions, day-to-day fluctuations in seabirds attendance due to the breeding cycle^47^, or within-night behavioural patterns, as a majority of Procellariiform seabirds tend to visit their nest soon after sunset^48^. To check if birds habituated to the light stimulus over the course of the night, we additionally investigated the effect of the duration of the experiment on the difference in the number of flying birds, and found no effect (Supplementary Table S4).

### The interval experiment

**Figure 4:**
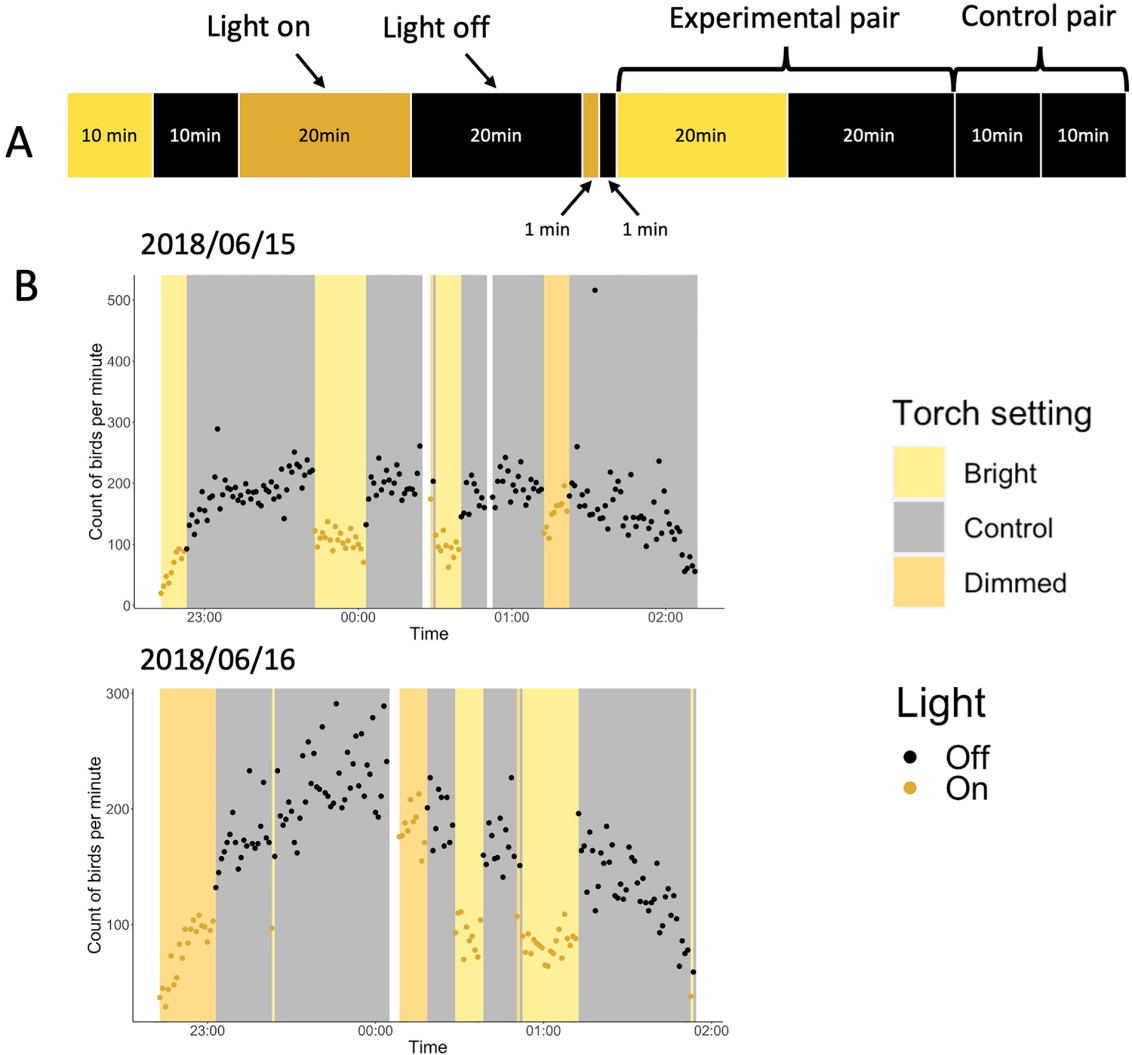
(**A**) Visualisation of treatment series of the interval experiment. Each segment corresponds to the colour (yellow = bright white, orange = dimmed white, black = light off) and duration for which the light was on (or off). In the experimental pairs, we turned the light on and off for different lengths of time (1-, 10- and 20-min intervals). In the control pairs we turned the light off for two consecutive intervals (‘light-off’, ‘light-off’). (**B**) Two example days, 15th and 16th June 2018, of the interval experiment. The graphs show the count of birds per minute against time. Different colour bars represent different settings (bright white, dimmed white and control) and the two dot colours represent light on (yellow) and off (black). White bars represent breaks in the experiment caused mainly by switching to a new recording or by technical mistakes.

**Figure 5:**
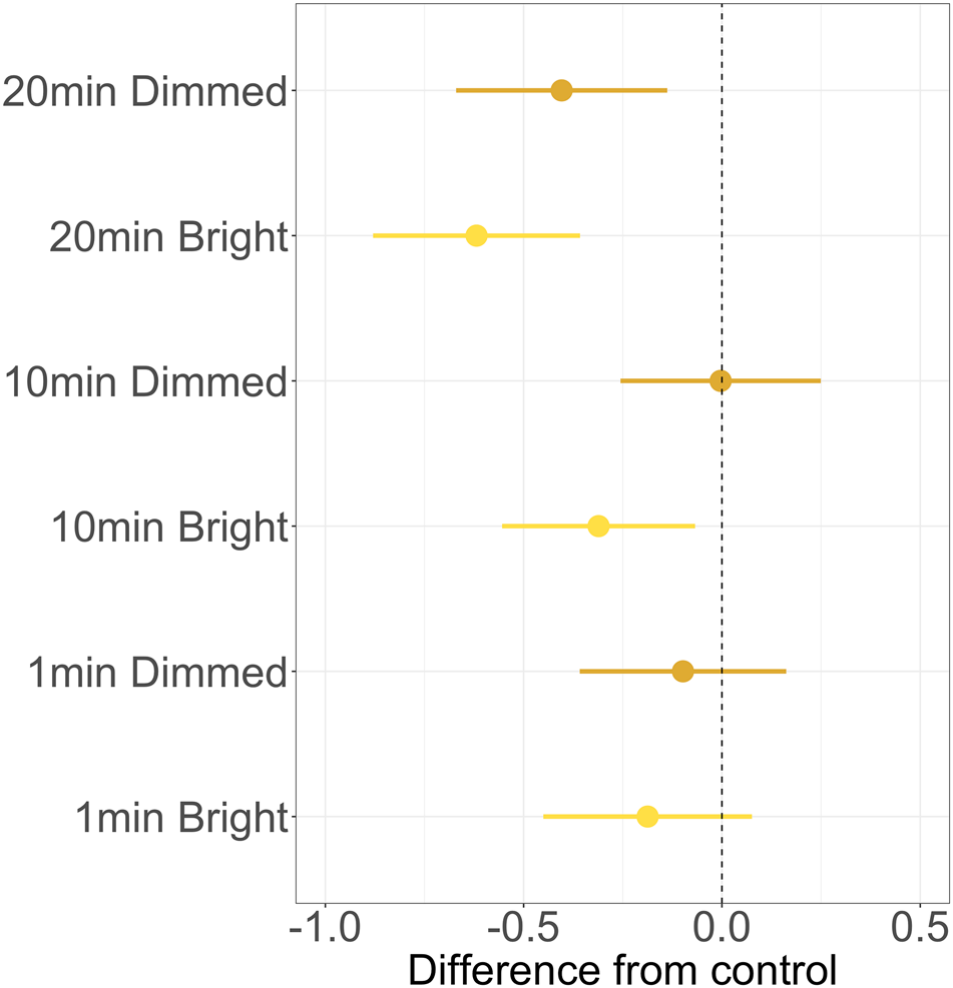
Results of post-hoc tests in the interval experiment. The graph shows the estimated coefficients and 95% confidence intervals of six experimental pairs when comparing the difference in ‘light-on’ vs ‘light-off’ to the control (‘light-off’ vs ‘light-off’, dashed line). It showed that both 20-min treatments, as well as the 10-min bright treatment, had a significant effect on the number of birds, whereas the 10-min dimmed treatment and both 1-min treatments did not. For detailed statistics, see Table 2.

In the second experiment, we switched on two intensities of broadband white light (dimmed or bright white light) for 1-, 10-, and 20-min intervals. We used a similar pairing structure of our treatments to the spectra experiment. This time, however, experimental pairs comprised of turning light on and off for two equal intervals (Fig. 4A), whereas, in control pairs, we kept the light off for two consecutive intervals. The data collection resulted in the following experimental pairs for durations of 1, 10, and 20 min, respectively: 11, 10 and 10 bright white pairs; 10, 9, 9 dimmed white pairs; and 8, 10 and 9 control pairs. To understand how different durations of ‘light-on’ treatments affected the number of flying shearwaters, we compared the difference in the average number of counted birds per minute in the experimental pairs (‘light-on’ vs. ‘light-off’) to the difference in the control pairs (‘light-off’ vs. ‘light-off’) using a post-hoc GAM. The model was well fitted with 95.3% of the deviance explained. We detected fewer flying shearwaters during ‘light-on’ versus ‘light-off’ periods during the 20-min intervals (for both the dimmed and bright light treatments), as well as the 10-min bright light treatment (Table 2, Figs. 4B, 5). There was a 46% (95% CI [30, 58]) decrease in counted birds when we turned on the bright white light for 20 min compared to the control pair (20 min ‘light-off’, 20 min ‘light-off’); a 33% (95% CI [13, 49]) decrease when we turned on the dimmed light for 20 min; and a 27% (95% CI [7, 42]) decrease when the bright light was turned on for 10 min.

**Table 2:** Summary of the results of post-hoc pairwise tests for the interval experiment, showing the comparison of differences in bird count between experimental pairs (‘light-on’, ‘light-off’) and control pairs (‘light-off’, ‘light-off’) of various durations.

Comparisons	Estimate	Odds Ratio	s.d	t ratio	*P*-value
1 min Control vs 1 min Bright	**− **0.187	0.829	0.132	**− **1.415	0.1605
1 min Control vs 1 min Dimmed	**− **0.098	0.906	0.131	**− **0.75	0.4554
1 min Dimmed vs 1 min Bright	**− **0.089	0.915	0.126	**− **0.71	0.4797
10 min Control vs 10 min Bright	**− **0.311	0.733	0.122	**− **2.54	0.0128
10 min Control vs 10 min Dimmed	**− **0.003	0.997	0.127	**− **0.026	0.9796
**10 min Dimmed vs 10 min Bright**	**− 0.308**	**0.735**	**0.129**	**− 2.378**	0.0196*
**20 min Control vs 20 min Bright**	**− 0.619**	**0.539**	**0.131**	**− 4.712**	< 0.0001***
**20 min Control vs 20 min Dimmed**	**− 0.404**	**0.668**	**0.134**	**− 3.017**	0.0033**
20 min Dimmed vs 20 min Bright	**− **0.215	0.807	0.135	**− **1.588	0.1159
1 min Bright vs 10 min Bright	**− **0.106	0.899	0.126	**− **0.844	0.4009
10 min Bright vs 20 min Bright	**− **0.19	0.827	0.128	**− **1.484	0.1415
**1 min Bright vs 20 min Bright**	**− 0.296**	**0.744**	**0.13**	**− 2.282**	0.0249*
1 min Dimmed vs 10 min Dimmed	0.112	1.119	0.129	0.872	0.3855
**10 min Dimmed vs 20 min Dimmed**	**− 0.283**	**0.754**	**0.135**	**− 2.089**	0.0397*
1 min Dimmed vs 20 min Dimmed	**− **0.171	0.843	0.132	**− **1.294	0.1991

Estimates taken from the GAM show log-transformed differences in counted birds. For example, 20-min bright white caused a (0.539–1) * 100% = **− **46.1% decrease in counted birds when we turned on the light compared to 20-min control pair (‘light-off’, ‘light-off’). Significant results are marked in bold. ***P < 0.001; **P < 0.01; *P < 0.05.

Furthermore, there was a lower average number of flying seabirds per minute in longer ‘light-on’ periods than shorter ones. This pattern, however, was only found in some tested pairs. Comparing durations of 1 and 20 min for bright white light, there was a 26% (95% CI [4, 42]) decrease in counted birds during the longer duration treatment. Similarly, comparing durations of 10 and 20 min for dimmed white light, there was a 25% (95% CI [2, 42]) decrease in counted birds during the longer duration treatment. In other words, in both the bright and dimmed treatments, there were fewer birds flying when we turned on the light for a longer period. The smoothed terms of time relative to midnight, as well as the random effects of pair and calendar day showed significant effects on the numbers of the birds counted (Supplementary Table S3).

## Discussion

Our results show that anthropogenic light impacted the number of nocturnally flying adult Manx shearwaters at their island breeding colony and that this effect varied with changes in the wavelength, brightness and duration of the light source. We counted fewer shearwaters in flight in the presence of an illuminated torch (flashlight), providing evidence for negative phototaxis. This is consistent with previous research, in which analysis of radar data revealed that some bird species avoid bright areas and aircraft lights while migrating ^24, 49, 50^. Another study reported that adult Scopoli’s shearwaters might be perturbed from provisioning their chicks due to an outdoor disco event^46^, although the effects of disturbance from high-intensity light and sound were not experimentally separated. Thus, to our knowledge, we provide the first experimental evidence that seabirds may be repelled by artificial light.

The first objective of our experiments was to identify whether seabirds may respond differently to red, green, blue, and white (dimmed and bright) lights. Based on previous studies, we expected to find a greater effect of light with increasing intensity and with shorter wavelengths. In accordance with our predictions, we observed a lower number of flying seabirds when using bright white light than dimmed white light. We also counted fewer seabirds when using short compared to long wavelengths, as Manx shearwaters were more repelled by green/blue than by red light. We expected this interaction between brightness and wavelength, since examination of the retina of closely related species revealed that diving seabirds are more sensitive to blue and green colours than to red, which is probably an adaptation to diving in sea water where the blue green wavelengths (deep sea blue 475 nm) are most effective for observing prey and predators underwater^40, 51^.

The second objective of our experiments was to identify whether longer durations of illumination would cause a stronger behavioural response. Previous research manipulating the duration of light compared lights that flashed 6–34 times per minute to continuous light and found that fewer birds were attracted towards flashing, short ‘light-on’ periods, than to continuous light^6, 9–11, 52^. We did not investigate flashing light, but we tested different durations of continuous light and found that longer light durations elicited stronger responses, resulting in fewer flying birds. In the bright white light treatment, however, we did not find a difference between two consecutive steps (1 min to 10 min and 10 min to 20 min), but only between 1 and 20 min, suggesting that the effect might be gradual. This pattern was not found for dimmed white light (the difference was only detected between 10 and 20 min) implying that the effect of duration of the ‘light-on’ treatment might differ depending on the characteristics of the light stimulus. Further studies would be needed to investigate the interaction between spectral composition and ‘light-on’ duration on avoidance behaviour in seabirds.

Our experiments demonstrate that adult Manx shearwaters are repelled by artificial light, in contrast to the apparent attraction of fledglings to light that causes them to ground in coastal towns^25^. This difference could potentially be caused by differences in the developmental stage of the birds’ eyes. It has been suggested that the eyes of young burrow-nesting seabirds, as well as hatchling marine sea turtles, are not fully developed upon leaving the nest, thus possibly resulting in attraction towards light^53, 54^. Avoidance of light may develop later over the animals’ lifetime and as their eyes finish development, but the mechanisms underlying this remain unclear. Adult Procellariiformes regularly avoid activity at their colonies during moonlit nights to decrease their chance of being depredated by diurnal avian predators that exploit bright conditions at night^41–44^, and this same avoidance response might be behind the lower number of seabirds flying during the ‘light-on’ stimuli during our experiments. Alternatively, adult seabirds might have avoided the light because it was a new, unknown stimulus near their nest (neophobia)^55, 56^. Our findings, however, indicate that light avoidance in Manx shearwaters is not site-specific as it occurred at two sites on Skomer Island, including one with no human access and one occasionally disturbed by human presence. There can be up to 30 people staying on the island overnight that are required to use dim red lights or red filters on torches if they walk around the colony at night. Other light pollution on Skomer Island comes from anchored vessels nearby and the costal developments 5 km away from the island. Further studies investigating the reaction of other adult Procellariiformes to light pollution would be useful to determine whether our findings apply to other species elsewhere and to uncover the mechanisms driving negative phototaxis in adult seabirds.

Nevertheless, our experiment found that adult Manx shearwaters avoid anthropogenic light at the colony and provided evidence that brighter light and shorter wavelengths (blue and green) are more repulsive. Such results should be taken into account when determining which types of light to use near Procellariiform breeding grounds, especially for light pollution that may unexpectedly appear near a colony, for example from people visiting the colony at night, cars driving past or vessels anchoring for the night. Decreasing light pollution by covering the upward spill of light, choosing longer wavelengths, or reducing the time that lights are on, have already been recommended for areas where Procellariiform fledglings ground^13, 25^. Our findings provide evidence for the same mitigation measures to be considered at or near breeding colonies of burrow-nesting nocturnal seabirds. We show that lights at or near the breeding colony can result in avoidance behaviour from adult Manx shearwaters. This could result in attendance to the burrow being perturbed^46^. As a result, we recommend that the use of lights in view of shearwater colonies, including those that appear infrequently, should be carefully considered, and if possible, lights should be reduced to a minimum or covered, for example, by using window blinds. Our results also suggest that if lights cannot be avoided, using long wavelength light, such as red-filtered light, should be preferred to short or broadband wavelengths. Indeed, we found that red light did not induce avoidance by shearwaters, compared to the same intensity of blue and green coloured lights. However, note that we only tested a single, relatively low intensity of monochromatic red light (4.3 W), so higher intensities may still result in avoidance behaviour. Further investigations into the effect of light pollution with higher intensity lights should therefore be considered.

Furthermore, some of our longer ‘light-on’ periods resulted in fewer flying shearwaters compared to short ones, suggesting that a shorter exposure to light can cause less disturbance to birds. Thus, mitigation measures that include on-demand streetlamps or obstruction lighting may lower the negative impact of light pollution. Altogether, our results support previous evidence that short wavelengths, long exposure to light and stronger light intensity seem to have a stronger effect on the behaviour and physiology of a range of species^6, 9, 10, 25, 28, 52, 57–60^. Thus, our results are likely to be applicable to many nocturnally active animals, although we also recognise that a taxon-specific approach is necessary when investigating the impact of light pollution on animals^25^.

Our research had some limitations including the fact that the light intensity of different colours (red, green or blue) was compared without controlling for the visual sensitivity of Manx shearwaters. Without a detailed understanding of the visual perception of Manx shearwaters it is hard to conclude whether birds were more influenced by a specific colour of light or if the decrease in the number of birds flying was caused by the higher perceived intensity of the light itself. Since birds were less repelled by red light on brighter nights, we do consider that darker nights created enough contrast for a bird to perceive the red light and thus induce some avoidance behaviour, therefore giving support to the latter explanation. For artificial light impact mitigation purposes, the reason why certain lights have more or less effect is of secondary importance, so our key result here is that red light caused less disturbance than green, blue, dimmed and bright white light. Nonetheless, the impact of background light created by celestial or human-made objects can have an impact on the effect of artificial light on birds^34, 61^, and thus further research should consider the role of background light on the perception and behaviour of animals towards light pollution. Furthermore, we found an effect of light on the number of flying Manx shearwaters when using 20 min (for both dimmed and bright light) and 10 min (bright light only) treatments. In contrast, having the light on for only one minute in the interval experiment did not show any effect regardless of intensity. Although this can initially appear to be at odds with the results of our spectra experiment where one-minute exposure to white light led to a reduction in the number of flying birds, this discrepancy could be due to the lower statistical power in our interval experiment, which may not have been sufficient to detect a significant difference in the one-minute treatment (n = 10 in the interval experiment compared to n = 86 in the spectra experiment for dimmed white). Therefore, we advise caution when interpreting this result and recommend that it is investigated in future with a larger sample size.

Finally, we compared our research to previous studies that investigated the effects of artificial light on the behaviour of birds with lights being constantly on ^24, 25, 49, 62, 63^ or turned off for a period of time (20 min in ^64^ or 135 s in ^45^). Even though the torch used in our experiment satisfied the definition of light pollution^1^, the light stimuli we produced differed from other light stimuli regularly encountered by Manx shearwaters (such as streetlights along the coast or illuminated vessels). However, our experiment did not aim to emulate all potential “natural” light pollution, which might require modifying and switching off infrastructures such as streetlamps or navigation buoys, which could be both impractical and possibly dangerous. Rather, we aimed to investigate the fundamental processes involved in the shearwaters’ response to light pollution and explore the effects of light characteristics on their behaviour. A key advantage of our designed experimental framework, unlike a “natural” set-up with existing lighting infrastructure, is that it allowed us to manipulate the characteristics of the light stimulus and disentangle these from potentially confounding variables such as sound, movement, or smell of human habitation. Further research should consider undertaking experiments that include manipulation of light commonly encountered by seabirds, such as streets lights or vessels lamps, to better understand the impact of light pollution on shearwaters and improve the light mitigation guidelines.

In conclusion, our finding of light avoidance behaviour in Procellariiform seabirds, rather than attraction to light, is indicative that we still understand little about how light impacts animals at different stages of life and of the annual cycle. The impact of light avoidance, unlike attraction, requires the use of devices to record animal movement, and thus is harder to detect since it does not lead to easily observable and measurable indicators such as congregation, grounding or collisions. Light could be utilized as a conservation measure for negatively phototactic animals, for example when used as a deterrent to ensure the safety of people and animals encountering ships or urban areas^65, 66^. Nevertheless, light avoidance can also have negative effects by altering and restricting animals’ movements, resulting in changes to the distribution of populations and potential lower individual fitness^66–68^. We therefore encourage more research examining the impact of light pollution on animals at various locales and during different life stages.

## Methods

### Study site and species

The Manx shearwater is a medium-sized Procellariiform seabird that mainly breeds on islands in the eastern North Atlantic between April and September. In autumn, young birds fledge at night, and this is when they are particularly susceptible to the impacts of artificial light^34^. Manx shearwater groundings are reported frequently close to colonies on the Canary Islands, Madeira and Azores^69^, in western Scotland^34, 36^, and around the mainland coast of southwest Wales, near large colonies of shearwaters located on Skomer and Skokholm Islands (Anna Sutcliffe pers. comm.).

Skomer Island (Pembrokeshire, southwest Wales, UK., 51˚ 44’ N, 5˚ 17’ W, Fig. 1A), where this study was undertaken, hosts the biggest colony of Manx shearwaters in the world, with around 317,000 breeding pairs^70^. There is some anthropogenic light from vessels and the costal developments 5 km away from Skomer Island, but very little anthropogenic light on the island itself, with a maximum of ~ 30 people staying on the island overnight. At night, staff and tourists use dim red lights or red filters on torches for observing seabirds and walking around the island.

### Experimental design

The experiment was undertaken over 20 days between 14th June and 14th August 2018. A Forward-Looking Infrared thermal camera (FLIR T620, Axsys Technologies, Rocky Hill, Connecticut, United States) with a frame rate of 18.84–20.6 Hz was used to record flying adult Manx shearwaters. Next to the camera, a T50 Waterproof LED handheld torch light (Icefire Lighting Ltd., Shen Zhen, China), similar to those used regularly by the staff and visitors, was positioned and covered with gel filters (Cokin, Rungis, France) to generate monochromatic blue, green, red and dimmed white light treatment (Fig. 1B, Supplementary Table S5). The torch light was positioned parallel to the ground and gave a wide beam of light (Supplementary Fig. S2). Due to the sensitivity to water damage of the thermal camera, the study was undertaken only on nights with little or no rain.

The light intensity of the torch light was measured using an OceanOptics USB2000 + fibre optic spectrometer, calibrated using an Oriel Instruments 6035 Hg (Ar) lamp (Fig. 1B). The central wavelength and bandwidths (Full-Width-At-Half-Maximum) when using the filters were 450 nm (18 nm), 540 nm (45 nm), and 620 nm (60 nm) for the blue, green, and red filters, respectively. The total measured signal can be integrated to estimate the radiant flux for each source. The flux for the torch (bright white) was 32 W and its colour temperature was estimated at 5175 K (Supplementary Table S5). The flux for the dimmed white was 3.3 W, whereas the flux was 4.3 W for the red filter, 2.0 W using the green filter, and 1.4 W using the blue filter. Therefore, by using the filters, the total flux of each light source was of the same order of magnitude, with an average value of (2.7 ± 1.3) W.

We performed two experiments (10 nights each, Supplementary Table S6) to investigate the influence of light on the number of flying shearwaters. The first experiment (the “spectra experiment”) assessed the effect of different spectra and intensities of light, whereas the second (the “interval experiment”) investigated the influence of different lighting durations and intensities. For both experiments, we recorded footage of shearwaters in flight in front of the camera throughout the experiment for later calculation of the number of flying shearwaters.

The spectra experiment used different monochromatic light — blue, green and red —with the intensity of 1.4 W, 2 W and 4.3 W respectively (Fig. 1B). We also used broadband white light of similar intensity (‘dimmed white’, 3.3 W) and a tenfold more intense broadband white light (‘bright white’, 32 W). The experiment was split into ‘control’ and ‘experimental’ pairs, which comprised two consecutive one-minute intervals (Fig. 2A). A one-minute interval was chosen because it is long enough for a bird 500 m away to respond (assuming a flight speed of 11 m/s ^71^), but short enough to allow switching between treatments across the experiment without biases caused by environmental changes in the colony (such as wind speed and direction or cloud cover). For control pairs, the light was kept off for both one-minute intervals, while for experimental pairs the light was switched on for the first minute, then off the second. This paired design further helped to account for variation in the number of birds in the colony over the course of the night. The design resulted in two explanatory variables: light (‘light-on’ and ‘light-off’) and setting (‘blue’, ‘green’, ‘red’, ‘dimmed white’, ‘bright white’ and ‘control’). The order in which control and experimental pairs were arranged was selected each day using a constrained randomised design: each of the six settings was used 10 times over two hours, and none of the settings was repeated more than twice in a row.

The experiment was undertaken at two locations on Skomer Island: one near the farmhouse, a location disturbed by the presence of tourists, and another on the Neck, an area that is not accessible to tourists (Fig. 1A). The torch and thermal camera were positioned next to the edge of a cliff facing the sea on the Neck, whereas near the farmhouse, the torch was placed on a hill facing land. The beam of the light was wide so that it lit the cliff edge and the ground in front of the torch (Supplementary Fig. S2). To control for night sky brightness, we used a Sky Quality Meter (SQM, Geoptik, Verona, Italy) to measure ambient light levels in magnitudes per square arc second (mag arcs^–2^). We measured the darkness of the night sky with the SQM directed upwards from a similar position and height (170 cm above ground) each night of the spectra experiment. Night darkness for each hour of the experiment was taken to be the mean between measurements taken at the beginning and at the end of the hour. As the original values ranged between 19.48–22.04, we rescaled those values so that the mean was zero to facilitate interpretation of model results (Supplementary Table S6).

The interval experiment involved turning on two intensities of broadband white light: ‘dimmed white’ and a tenfold more intense ‘bright white’ light for 1-, 10- and 20-min intervals (Fig. 4A). We used a similar pairing structure for our treatments as in the spectra experiment, in which experimental pairs comprised two consecutive intervals of equal duration (1, 10, or 20 min). In control pairs, the light was kept off for both intervals. In experimental pairs, the light was switched on for the first interval and switched off for the second. This resulted in three explanatory variables: interval duration (1, 10, 20 min), setting (‘dimmed white’, ‘bright white’ and ‘control’) and light (‘light-on’ and ‘light-off’). The order of experimental and control pairs was selected every day using a constrained randomised design; each of the six combinations (setting × interval duration) was used once per night. Due to time constraints the interval experiment was limited to one location (the Neck) and nights with no visible moon as we expected that background light might affect the response of flying seabirds towards the experimental stimulus^34, 61^.

### Statistical analysis

We counted the number of birds in flight in the videos recorded by the thermal camera using the Motion-Based Multiple Object Tracking module in MATLAB (R2017a, MathWorks Inc.), which tracks moving objects in two dimensions. The parameters were set to track objects bigger than 20 pixels and smaller than 4000 pixels, as the module performed well with those parameters upon a visual inspection. This threshold was set to recognise only birds that were 5–85 m from the camera. To validate this method, birds were manually counted in 5-min samples (with a start point generated at random) of each c.1-h video that was run through the software, for a total of 4 h out of the 47 h 28 min of footage. We found that the counts performed by the module and manual counts were highly correlated (Pearson’s Correlation test sample estimates 96.72% ± 0.93%, t_238_ = 58.791, *P* < 0.001, Supplementary Fig. S1).

Analyses were conducted in R (version 4.0.2, R Core Team 2020). The package ‘mgcv’^72^ was used to construct generalised additive models (GAMs) with log link and negative binomial error distributions. The model assessed whether the number of birds differed between treatments while accounting for seasonal and within-night variation in colony attendance.

For the spectra experiment, we fitted a model with the following formula to the data:

$${\text{Birds}}\_{\text{count}}\sim {\text{Setting}}*{\text{Light}}*{\text{Location}} + {\text{Setting}}*{\text{Light}}*{\text{Night}}\_{\text{Darkness}} + \left( {{\text{random}} = {\text{Pair}}} \right) + \left( {{\text{random}} = {\text{Day}}} \right) + {\text{s}}\left( {{\text{Time}}} \right)$$

In this model, the response variable was the number of counted birds per minute and the explanatory variables were categorical factors of setting, on/off light and location (the Neck/the Farm) and the continuous variable of night darkness. Our model assumed that an individual bird passed only once in front of the camera during a trial, but it remains a possibility that some birds passed multiple times. The night darkness measurement was rescaled and varied between **− **1.3 (bright night) to 1.25 (dark night). We included a smoothed term of time relative to midnight (‘Time’) to account for non-linear variation in bird densities throughout the night unrelated to treatment (e.g. due to weather factors and within-night behavioural patterns^48^), specified as a thin plate regression spline with basis dimension chosen automatically. This variable additionally served to account for temporal autocorrelation arising from trials occurring close together in time. Julian date (‘Day’) was included as a random term to account for any changes caused by differences in weather between days. Additionally, a variable “Pair”, which assigned a consecutive number to each experimental and control pair, was included as a random term in the model to reflect the paired design of the experiment. To deal with overdispersion in our count response variable, we fitted a negative binomial error distribution.

We tested our hypotheses using post-hoc contrasts designed with the ‘emmeans’ package^73^. Specifically, we designed post-hoc tests to compare the difference in bird count between the two parts of each experimental pair (‘light-on’ vs. ‘light-off’) with the control pair difference (‘light-off’ vs. ‘light-off’), as well as between different experimental pairs. In other words, we compared the difference in counted birds of each experimental pair (e.g. blue light vs. ‘light-off’) with a control pair (‘light-off’ vs. ‘light-off’) and other experimental pairs (e.g. red light vs. ‘light-off’). We also tested whether location had an effect on the difference in experimental pairs of the same setting (e.g. green light vs. ‘light-off’ on the Neck comparing to green light vs. ‘light-off’ at the Farm) and if night darkness had an effect on the difference in experimental pairs compared to control pairs. All p-values were adjusted with a Tukey correction for multiple post-hoc testing. We additionally explored the potential habituation of birds towards the light stimulus over the course of the night, by testing the effect of the time from start of the experiment on the difference in the number of flying birds (Supplementary Table S4).

We analysed the interval experiment using GAMs with a log link function and negative binomial error distribution*.* This time we fitted a model with the following formula to the data:

$${\text{Mean}}\_{\text{of}}\_{\text{birds}}\_{\text{count}}\sim {\text{Setting}}*{\text{Light}}*{\text{Interval}}\_{\text{Duration}} + \left( {{\text{random}} = {\text{Pair}}} \right) + \left( {{\text{random}} = {\text{Day}}} \right) + {\text{s}}\left( {{\text{Time}}} \right)$$

In this model, the response variable was an average number of counted birds per minute across each interval. The explanatory variables were three categorical factors of setting, on/off light and the interval duration (1, 10, 20 min). Similar to the spectra experiment, we used a smooth term of the time relative to midnight, and random terms of “pair” and Julian date. The ‘emmeans’ package was used to compare differences between the experimental pairs and the control pairs for each interval duration separately. Specifically, we compared the difference in the average number of counted birds in each experimental pair (e.g. bright white 10 min vs 10 min light off) with a control pair of the same duration (e.g. 10 min light off vs 10 min light off). We additionally investigated if longer durations of light resulted in lower average number of counted birds per minute by comparing different durations of experimental pairs (e.g. we compared the difference between bright light 1 min vs 1 min light off to bright light 10 min vs 10 min light off). All the p-values were adjusted with a Tukey correction for multiple post-hoc testing.

### Ethical note

All methods and procedures adhere to ASAB/ABS Guidelines for the Use of Animals in Research and the work was conducted after ethical approval by Islands Conservation Advisory Committee and the Local Ethical Review Process of the University of Oxford. The reporting follows the recommendations in the ARRIVE guidelines.

## Supplementary Information

Supplementary Information.
