# Supplementary material for: Avoidance of different durations, colours and intensities of artificial light by adult seabirds
Source: Sci Rep. 2021 Sep 23;11:18941. doi: 10.1038/s41598-021-97986-x (PMC8460786; doi:10.1038/s41598-021-97986-x)
Supplement: Supplementary file 1 — Supplementary Information. [file 41598_2021_97986_MOESM1_ESM.docx]

# Supplementary Materials: Avoidance of different durations, colours and intensities of artificial light by adult seabirds

Martyna Syposz^1*^, Oliver Padget^1^, Jay Willis^1^, Benjamin M. Van Doren^1^, Natasha Gillies^1^, Annette L. Fayet^1^, Matt J. Wood^2^, Aarón Alejo^3^ and Tim Guilford^1^

^1^ Department of Zoology, University of Oxford, Mansfield Road, Oxford OX1 3SZ, UK

^2^ School of Natural & Social Sciences, University of Gloucestershire, Francis Close Hall, Cheltenham, GL50 4AZ, UK

^3^ Department of Physics, University of Oxford, South Parks Road, Oxford OX1 3PS, UK

* author for correspondence: [syposzmartyna@gmail.com](mailto:syposzmartyna@gmail.com)


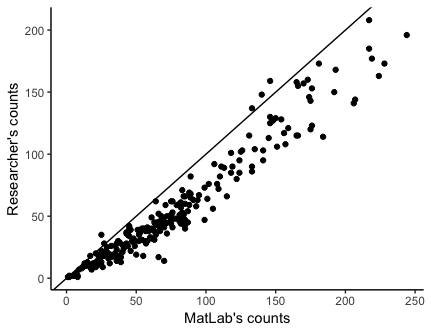


**Supplementary Figure S1.** Results from manual counts of birds in 5 min subset of each hour-long video to validate the Motion-Based Multiple Object Tracking module in MatLab. The count of birds performed by the module and manual counts were highly correlated (the Pearson Correlation test sample estimates was 96.72% ± 0.93%, t_238_ =58.791, p < 0.001).

**Supplementary Table S1.** Summary of the results of post-hoc tests showing the difference between the two locations at the Neck and the Farm. Estimates represent log-transformed differences in bird counts between light on and off of experimental and control pairs compared between two locations ***P < 0.001; **P < 0.01; *P < 0.05.

| Comparisons | Estimate | Odds  Ratio | s.d. | t ratio | p-value |
| --- | --- | --- | --- | --- | --- |
| Control: Farm vs Neck | 0.0004 | 1 | 0.082 | 0.005 | 1 |
| Bright white: Farm vs Neck | 0.238 | 1.269 | 0.087 | 2.746 | 0.0679 |
| Dimmed white: Farm vs Neck | 0.177 | 1.194 | 0.083 | 2.139 | 0.2688 |
| Blue: Farm vs Neck | 0.154 | 1.166 | 0.085 | 1.819 | 0.4539 |
| Green: Farm vs Neck | 0.107 | 1.113 | 0.081 | 1.32 | 0.7740 |
| Red: Farm vs Neck | 0.15 | 1.162 | 0.086 | -1.735 | 0.5091 |

**Supplementary Table S2.** Summary of the results of post-hoc tests showing the effect of night darkness on the difference in bird counts in experimental pairs (light on vs light off) compared to control pairs (light off vs light off). Estimates represent log-transformed differences in counted birds between light on and off. Results show how red colour caused a (0.778 - 1) *100% = -22.2% decrease in the number of flying birds for every 1 unit increase when the light was turned on during dark nights compared to moonlit nights. ***P < 0.001; **P < 0.01; *P < 0.05.

| Comparisons | Estimate | Odds Ratio | s.d. | t ratio | p-value |
| --- | --- | --- | --- | --- | --- |
| Control vs Bright | -0.171 | 0.843 | 0.094 | -1.820 | 0.0693 |
| Control vs Dimmed | 0.045 | 1.046 | 0.09 | 0.496 | 0.6203 |
| Control vs Blue | -0.112 | 0.894 | 0.092 | -1.221 | 0.2224 |
| Control vs Green | -0.105 | 0.9 | 0.091 | -1.149 | 0.2508 |
| Control vs Red | -0.251 | 0.778 | 0.096 | -2.610 | 0.0093 ** |

**Supplementary Table S3.** Summary of the smooth and random terms of GAMs for the spectra and interval experiments. ***P < 0.001; **P < 0.01; *P < 0.05.

| **Random and smooth terms of GAMs for the spectra exp.** | edf | Ref.df | Chi.sq | p-value |
| --- | --- | --- | --- | --- |
| s(Pair) | 365.36 | 495 | 2031.8 | < 0.0001 *** |
| s(Calendar day) | 7.89 | 8 | 11616.68 | < 0.0001 *** |
| s(Time relative to midnight) | 5.937 | 6.22 | 74.94 | < 0.0001 *** |
| **Random and smooth terms of GAMs for the interval exp.** |  |  |  |  |
| s(Pair) | 52.272 | 77 | 267.12 | < 0.0001 *** |
| s(Calendar day) | 8.691 | 9 | 4872.08 | < 0.0001 *** |
| s(Time relative to midnight) | 5.518 | 6.354 | 33.26 | < 0.0001 *** |

**Supplementary Note S1.** To investigate whether there is an effect of the duration of the experiment on the number of flying birds we added an additional interaction to the original model. The interaction was between setting, light and a continuous variable, Time_fromStart, calculated in minutes from the beginning of each experimental day.

Birds_count ~ Setting * Light * Location +

Setting * Light * Night_Darkness +

Setting * Light * Time_fromStart +

(random = Pair) + (random = Day) + s(Time)

We then run a post-hoc test to check if time from start of the experiment had an effect on the difference in experimental pairs comparing to control pairs. We found that the time did not have an effect on any of the pairs of light on and off (Table S4).

**Supplementary Table S4.** Summary of the results of post-hoc tests investigating the effect of duration of the experiment on the difference in bird counts in experimental pairs (light on vs light off) compared to control pairs (light off vs light off). Estimates represent log-transformed differences in counted birds between light on and off.

| Comparisons | Estimate | s.d. | t ratio | p-value |
| --- | --- | --- | --- | --- |
| Control vs Bright | 0.00072 | 0.001 | 0.49 | 0.624 |
| Control vs Dimmed | -0.00147 | 0.001 | -0.987 | 0.324 |
| Control vs Blue | 0.00002 | 0.001 | 0.013 | 0.99 |
| Control vs Green | 0.00003 | 0.002 | 0.017 | 0.986 |
| Control vs Red | -0.00067 | 0.001 | -0.456 | 0.648 |

**Supplementary Table S5.** The Cokin gel filters used during the experiment to create different wavelength and intensity of the light. The colour temperature of the source can be estimated from the light spectrum shown in Fig. 1B. Considering a weighted-average value for the wavelength of 560nm, the corresponding colour temperature of the source can be calculated using Wien’s law^1^ to be 5175K.

| **Settings** | **Filter type** |
| --- | --- |
| **Control** | None |
| **Bright white** | None |
| **Dimmed white** | 210 + 209 x 2 |
| **Blue** | 721 + 209 |
| **Green** | 139 + 298 |
| **Red** | 160 |


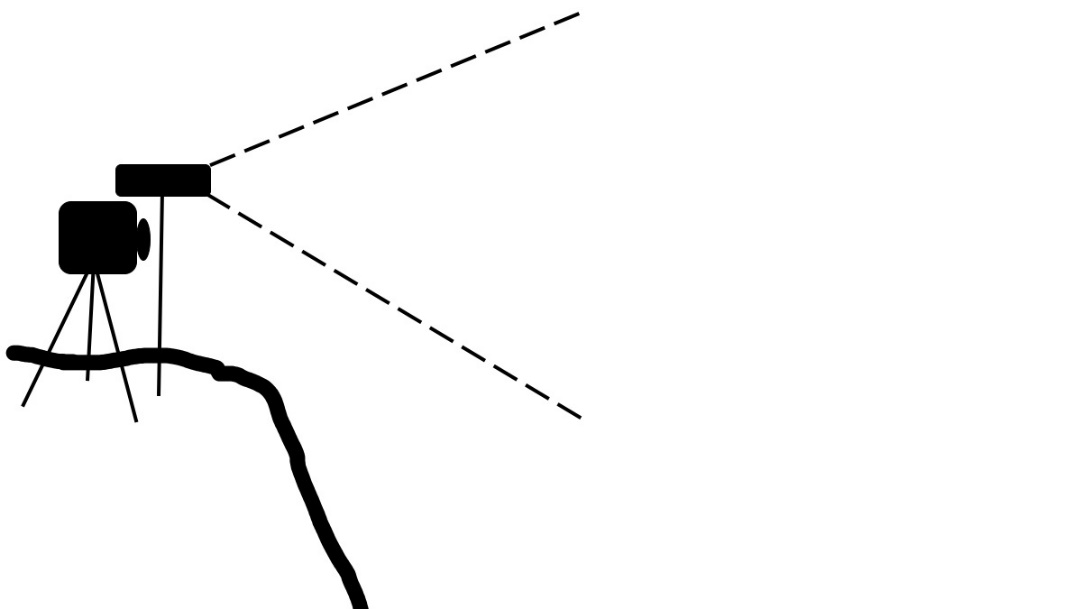


**Supplementary Figure S2.** A scheme of the experimental set up. The torch stood next to the thermal camera and both were facing horizontally. On the Neck the devices were positioned near to the edge of a cliff, facing the sea. Near to the farmhouse the torch and the camera were positioned on a hill, facing a flat field.

**Supplementary Table S6.** The spectra and interval experiments were undertaken over 10 days each. The sky quality measurements (SQM) are presented as a mean for each day of the spectra experiment. The SQM measurement were rescaled for purpose of the analysis.

| **Date** | **Experiment** | **SQM** | **SQM rescaled** |
| --- | --- | --- | --- |
| **14/06/2018** | Interval experiment | NA | NA |
| **15/06/2018** | Interval experiment | NA | NA |
| **16/06/2018** | Interval experiment | NA | NA |
| **17/06/2018** | Interval experiment | NA | NA |
| **18/06/2018** | Interval experiment | NA | NA |
| **19/06/2018** | Spectra experiment (near to farmhouse) | 20.5 | -0.27 |
| **20/06/2018** | Spectra experiment (near to farmhouse) | 20.4 | -0.43 |
| **17/07/2018** | Spectra experiment (the Neck) | 21.1 | 0.29 |
| **18/07/2018** | Interval experiment | NA | NA |
| **19/07/2018** | Spectra experiment (near to farmhouse) | 21.4 | 0.66 |
| **21/07/2018** | Spectra experiment (the Neck) | 19.8 | -1.03 |
| **23/07/2018** | Spectra experiment (the Neck) | 20.0 | -0.77 |
| **02/08/2018** | Spectra experiment (near to farmhouse) | 21.3 | 0.54 |
| **03/08/2018** | Spectra experiment (the Neck) | 21.2 | 0.42 |
| **04/08/2018** | Spectra experiment (the Neck). – one hour | 20.3 | -0.53 |
| **05/08/2018** | Spectra experiment (the Neck) – one hour | 22 | 1.25 |
| **10/08/2018** | Interval experiment | NA | NA |
| **12/08/2018** | Interval experiment | NA | NA |
| **13/08/2018** | Interval experiment | NA | NA |
| **14/08/2018** | Interval experiment | NA | NA |

**References**

1. NIST. CODATA VALUE: Wien Wavelength Displacement Law Constant. https://physics.nist.gov/cgi-bin/cuu/Value?bwien.
